# Diagnostic Yield and Clinical Impact of Trichoscopy in Alopecia: A Cross-Sectional Study From a Rural Tertiary Care Centre

**DOI:** 10.7759/cureus.113387

**Published:** 2026-07-26

**Authors:** Harshini Jagalur, Yogesh Devaraj, Mukunda R Swaroop, Shradha Gurudev, Lakshmi G Nagaraj, Ranjeeta S Chauwan, Apoorva S., Avani Thakur

**Affiliations:** 1 Dermatology, Adichunchanagiri Institute of Medical Sciences, Adichunchanagiri University, Mandya, IND; 2 Dermatology, Akash Institute of Medical Sciences, Bangalore, IND

**Keywords:** alopecia, alopecia areata, diagnostic yield, non-invasive diagnosis, non-scarring alopecia, rural dermatology, scarring alopecia, tinea capitis, trichoscopy

## Abstract

Background and objective

Alopecia encompasses a heterogeneous group of scarring and non-scarring disorders that often have overlapping clinical features, complicating diagnosis when scalp biopsy is unavailable. The real-world diagnostic yield of trichoscopy in rural Indian populations remains incompletely characterised. The objective of this study is to evaluate the diagnostic yield of trichoscopy, quantify its capacity to alter or refine provisional clinical diagnoses, and assess its role in reducing invasive investigations.

Methods

A hospital-based cross-sectional study enrolled 200 consecutive alopecia patients at a rural tertiary care centre over 18 months (April 1, 2024, to October 1, 2025). Trichoscopy was performed with a handheld dermatoscope (Illuco IDS-1100; ILLUCO Co., Ltd., Gyeonggi-do, Republic of Korea). A provisional diagnosis was recorded prior to trichoscopy; outcome was classified as confirmed, refined, or inconclusive against the final composite diagnosis. Discriminatory features were analysed by chi-square and Fisher's exact tests.

Results

Trichoscopy was diagnostically contributory in 195/200 (97.5%): confirming the diagnosis in 190/200 (95.0%), refining it in 5/200 (2.5%), and being inconclusive in 5/200 (2.5%). Two cases (2/200, 1.0%) of presumed alopecia areata were re-diagnosed as tinea capitis on identification of comma hairs, averting inappropriate corticosteroid therapy; three cases (3/200, 1.5%) of presumed telogen effluvium were re-diagnosed as female pattern hair loss based on hair diameter diversity >20%. Comma hairs demonstrated 78.6% sensitivity (11/14) and 100% specificity (186/186) for tinea capitis (p < 0.001), directed targeted KOH testing (12/14, 85.7% positive yield), and limited the diagnosis-deciding scalp biopsy rate to 2/200 (1.0%).

Conclusions

Trichoscopy shows strong potential as a first-line, non-invasive triage tool in routine dermatology practice, particularly in resource-limited settings where it may help confirm diagnoses, avert mismanagement, and reduce the need for invasive investigations; multi-centre validation against an independent reference standard is needed before this can be firmly established.

## Introduction

Alopecia is one of the most frequent dermatological complaints worldwide and encompasses a heterogeneous group of disorders broadly classified into non-scarring (non-cicatricial) and scarring (cicatricial) categories [1]. Despite differing pathogenic mechanisms, many alopecias share overlapping clinical phenotypes: patchy hair loss may equally represent alopecia areata, tinea capitis, trichotillomania, or early lichen planopilaris, while diffuse loss may reflect telogen effluvium, anagen effluvium, or early female pattern hair loss [2,3]. Misclassification at this stage carries real clinical costs: inappropriate corticosteroid therapy in undiagnosed tinea capitis (the so-called "tinea incognito" effect), delay in initiating anti-androgen therapy in early female pattern hair loss, and progression to irreversible scarring when cicatricial alopecias are recognised late [4,5].

Scalp biopsy remains the diagnostic gold standard in many ambiguous cases, but it is invasive, often unavailable in primary and secondary centres, and culturally less acceptable in many rural Indian populations [6]. Trichoscopy - videodermoscopy or handheld dermoscopy of the scalp and hair shafts - has emerged over the past two decades as a rapid, non-invasive, and inexpensive bedside extension of clinical examination [7,8]. By rendering follicular openings, hair shaft morphology, perifollicular skin, and vascular structures visible at 10-20× magnification, it allows the clinician to objectively identify pathognomonic markers such as comma hairs (tinea capitis), exclamation mark hairs (alopecia areata), hair diameter diversity (androgenetic alopecia), and loss of follicular openings (cicatricial alopecia) [9,10].

Although several Indian and international studies have catalogued trichoscopic features in individual disease categories, fewer have prospectively quantified the proportion of cases in which trichoscopy actually changes management - that is, the rate at which it confirms, alters, or fails to clarify the provisional clinical diagnosis [11-13]. Such data are particularly critical for rural tertiary care settings, where delayed diagnoses carry higher morbidity due to restricted access to histopathology, mycology, and specialist trichology services. Establishing the diagnostic yield of trichoscopy in these resource-limited environments is essential to justify its integration as a cost-effective, first-line triage tool. We therefore designed this cross-sectional study with the primary aim of determining the diagnostic yield of trichoscopy in unselected patients presenting with alopecia and the secondary aims of quantifying its reclassification impact, its ability to direct confirmatory investigations, and its consequent reduction in scalp biopsy requirements.

## Materials and methods

Study design and setting

This was a hospital-based, cross-sectional, descriptive study conducted in the Department of Dermatology, Venereology and Leprosy, Adichunchanagiri Institute of Medical Sciences, B.G. Nagara, Mandya district, Karnataka - a tertiary care teaching hospital serving a predominantly rural population in South India. The study was conducted over an 18-month period, from April 1, 2024, to October 1, 2025. Approval was obtained from the Institutional Ethics Committee, Adichunchanagiri Institute of Medical Sciences (approval No. AIMS/IEC/030/2024; dated April 1, 2024), prior to initiation, and the study was conducted in accordance with the Declaration of Helsinki. Written informed consent was obtained from all adult participants and from a parent or legal guardian for participants under 18 years of age. The study is reported in accordance with the STROBE (Strengthening the Reporting of Observational Studies in Epidemiology) statement for cross-sectional studies.

Sample size and selection

A consecutive sampling strategy was used. Assuming an expected diagnostic contribution of trichoscopy of approximately 90% based on previous literature [11], with 5% absolute precision and a 95% confidence level, the minimum required sample size was calculated as 139 patients. To allow meaningful subgroup analysis across diagnostic categories and based on the previous year's outpatient statistics (approximately 160 alopecia consultations per annum), a sample size of 200 was finalised for the 18-month study window. Inclusion criteria were: (i) patients of any age presenting with scarring or non-scarring alopecia, and (ii) willingness to provide informed consent. Exclusion criteria were: (i) patients refusing consent, (ii) uncooperative paediatric patients, and (iii) patients with active secondary bacterial infection of the alopecic patch precluding meaningful trichoscopic examination.

Clinical evaluation

A structured pre-designed proforma was used to record demographic data, duration and pattern of hair loss, precipitating factors, family and drug history, hair-care practices, systemic comorbidities, and cutaneous associations. General and dermatological examination included assessment of the pattern of hair loss (patterned/patchy/diffuse), regional distribution, hair pull test, and cutaneous markers of hyperandrogenism. Disease-specific severity was graded using established systems: Norwood-Hamilton and BASP (Basic and Specific) for male androgenetic alopecia - BASP separately documents the basic pattern of hairline recession (types L, M, C, and U, based on hairline shape) and the specific density/severity of thinning (types F, V, and C); Ludwig, Olsen, and Sinclair for female pattern hair loss; and the SALT (Severity of Alopecia Tool) score for alopecia areata, calculated as the percentage of scalp surface area affected, with the scalp divided into four regions (vertex, right profile, left profile, and posterior), each weighted according to its proportional surface area, yielding a total score from 0 to 100. A provisional clinical diagnosis was recorded for each patient before trichoscopy was performed.

Trichoscopic evaluation

Trichoscopy was performed using a handheld polarised dermatoscope (Illuco IDS-1100, 10× magnification; ILLUCO Co., Ltd., Gyeonggi-do, Republic of Korea) on dry scalp without immersion fluid. All trichoscopic examinations were performed and interpreted by a single dermatologist experienced in trichoscopy, who was also the clinician recording the provisional diagnosis; formal blinding of the examiner to the provisional diagnosis was not undertaken, as this was considered impractical in the real-world outpatient setting, and inter-observer agreement was therefore not assessed. Examination covered the affected area, the margin of the lesion, and the contralateral or unaffected scalp for comparison. Follicular pattern (yellow, black, red, white, and blue-grey dots; keratotic plugs; loss of follicular openings), interfollicular and perifollicular pattern (honeycomb pigmentation, perifollicular erythema, scaling, hair casts, and vascular changes), hair shaft morphology (miniaturisation, hair diameter diversity >20%, short vellus hairs, exclamation mark hairs, broken hairs, comma hairs, corkscrew hairs, tufted hairs, and Pohl-Pinkus constrictions), and specific signs (V-sign and starburst sign) were systematically documented. Hair diameter diversity was assessed over the frontal/frontoparietal scalp: a representative trichoscopic field with clearly visible hair shafts was selected, and all hair shafts within that field were visually compared for calibre, with a positive result defined as ≥20% of hairs showing a visibly reduced shaft diameter relative to adjacent terminal hairs. Photographs were obtained and stored under restricted access.

Confirmatory investigations and final diagnosis

Confirmatory investigations were performed selectively, guided by clinical and trichoscopic findings: complete haemogram and serum ferritin in suspected telogen effluvium; thyroid function tests in suspected diffuse hair loss with endocrine features; hormonal profile in suspected female pattern hair loss with hyperandrogenism; KOH (potassium hydroxide) mount in any patient with comma hairs, corkscrew hairs, or other suggestion of fungal infection; antinuclear antibody (ANA) profile in suspected discoid lupus erythematosus; and scalp biopsy, reserved for cases that remained diagnostically uncertain after non-invasive evaluation. The final diagnosis for each patient was the composite diagnosis after integrating clinical, trichoscopic, and laboratory or histopathological data.

Outcome definitions

The trichoscopic outcome was prospectively assigned to one of three mutually exclusive categories against the final composite diagnosis: (i) Confirmed - trichoscopic findings supported the provisional clinical diagnosis without altering it. (ii) Altered or refined - trichoscopic findings produced a change in the diagnostic category that was subsequently validated by the final diagnosis (e.g., suspected alopecia areata reclassified as tinea capitis after identifying comma hairs and confirmed on KOH). (iii) Inconclusive - trichoscopic findings were non-discriminatory between two or more diagnostic possibilities.

Diagnostic yield was defined as the proportion of patients in whom trichoscopy either confirmed or altered/refined the diagnosis (i.e., contributed actively to the final diagnosis).

Statistical analysis

Data were entered in Microsoft Excel (Microsoft Corporation, Redmond, WA, USA) and analysed using Epi Info version 7.2.5.0 (Centers for Disease Control and Prevention, Atlanta, GA, USA). Categorical variables are reported as frequencies and percentages, and continuous variables as mean ± standard deviation. The chi-square test was used to compare the frequency of trichoscopic features across diagnostic categories. A p-value < 0.05 was considered statistically significant. For the prespecified diagnostic-test analysis of comma hairs against the final diagnosis of tinea capitis, a 2 × 2 contingency table was constructed, and sensitivity, specificity, positive and negative predictive values, diagnostic accuracy, and likelihood ratios were calculated, each with 95% confidence intervals (Wilson score method). Statistical association was tested using Fisher's exact test owing to small expected cell counts.

## Results

Cohort overview

Of the 200 enrolled patients, 111/200 (55.5%) were male and 89/200 (44.5%) were female; the mean age was 33.40 ± 13.17 years. Non-scarring alopecias accounted for 193/200 (96.5%) of cases and scarring alopecias for 7/200 (3.5%). The most frequent diagnoses were male androgenetic alopecia (69/200, 34.5%), alopecia areata (40/200, 20.0%), female pattern hair loss (34/200, 17.0%), telogen effluvium (30/200, 15.0%), tinea capitis (14/200, 7.0%), trichotillomania (3/200, 1.5%), traction alopecia (2/200, 1.0%), and anagen effluvium (1/200, 0.5%); cicatricial cases comprised lichen planopilaris (3/200, 1.5%), discoid lupus erythematosus (2/200, 1.0%), pseudopelade of Brocq (1/200, 0.5%), and folliculitis decalvans (1/200, 0.5%).

Diagnostic outcome of trichoscopy

Of the 200 patients, trichoscopy confirmed the provisional clinical diagnosis in 190/200 (95.0%), altered or refined it in 5/200 (2.5%), and was inconclusive in 5/200 (2.5%). Overall diagnostic contribution - the primary outcome - was 195/200 (97.5%) (Table 1).

**Table 1:** Overall diagnostic outcome of Trichoscopy in 200 patients with alopecia. This table summarises how trichoscopic findings related to the final composite diagnosis across the whole cohort. Each patient was assigned to one mutually exclusive category - confirmed, altered/refined, or inconclusive. The diagnostically contributory total (195/200, 97.5%) is the sum of confirmed and altered/refined cases and represents the study's primary outcome. n: number of patients

Trichoscopy outcome	n	%
Confirmed clinical diagnosis	190	95.0
Altered or refined diagnosis	5	2.5
Inconclusive	5	2.5
Total diagnostically contributory	195	97.5
Total	200	100.0

Clinical impact of reclassification

Trichoscopy altered the provisional clinical diagnosis in 5/200 patients (2.5%), and in every instance, the change was clinically meaningful (Table 2). In two of these patients (2/200, 1.0%), a provisional clinical diagnosis of alopecia areata was revised to tinea capitis following trichoscopic identification of comma hairs and corkscrew hairs, subsequently confirmed on KOH mount; this reclassification averted inappropriate intralesional or topical corticosteroid therapy. In the remaining three patients (3/200, 1.5%), a provisional diagnosis of telogen effluvium was revised to female pattern hair loss once trichoscopy demonstrated hair diameter diversity >20%, redirecting management away from reassurance towards anti-androgen and minoxidil therapy. Reclassification from telogen effluvium to female pattern hair loss was based on the overall trichoscopic pattern (increased hair diameter diversity alongside frontal predominance of miniaturised/vellus hairs), with hair diameter diversity ≥20% as the decisive discriminating feature within this composite picture rather than as an isolated criterion.

**Table 2:** Cases in which trichoscopy altered the provisional clinical diagnosis (n = 5). This table details the five patients whose provisional clinical diagnosis was changed by trichoscopy, listing the pre-trichoscopy diagnosis, the decisive trichoscopic finding, and the validated final diagnosis. Each reclassification was management-changing. KOH: potassium hydroxide mount; n: number of patients

Provisional clinical diagnosis	Decisive trichoscopic finding	Final diagnosis	n
Alopecia areata	Comma hairs ± corkscrew hairs (KOH-confirmed)	Tinea capitis	2
Telogen effluvium	Hair diameter diversity > 20%	Female pattern hair loss	3

Inconclusive cases

Trichoscopy was inconclusive in 5/200 patients (2.5%). Three cases were overlapping presentations of female pattern hair loss with chronic telogen effluvium in which hair diameter diversity had not yet reached the 20% threshold; one was an anagen effluvium in which features overlapped with severe alopecia areata; and one was a cicatricial alopecia in which the trichoscopic features did not allow a confident distinction between lichen planopilaris and another scarring entity requiring histopathological correlation.

Discriminatory value of key trichoscopic signs

**Figure 1:**
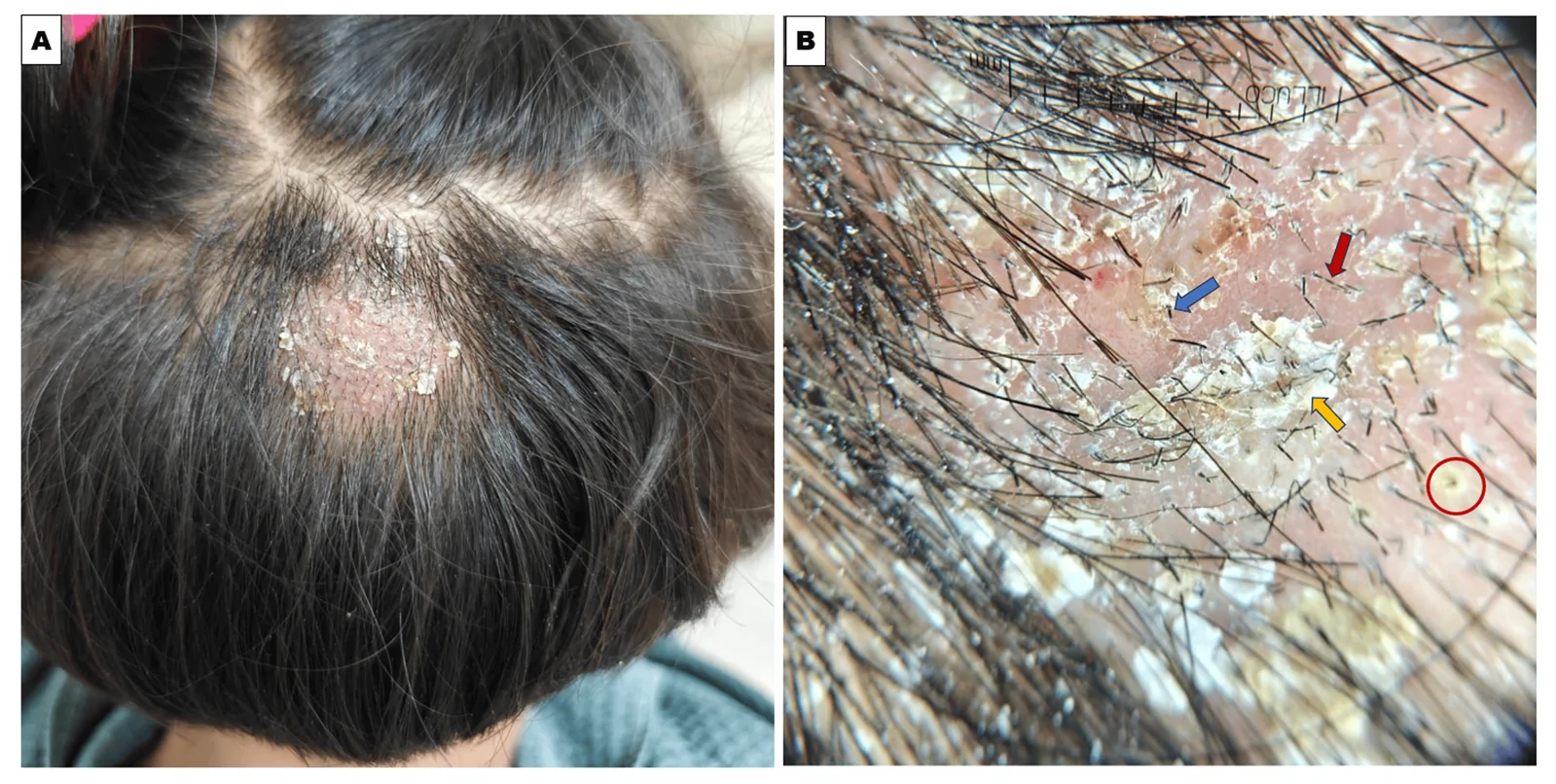
Tinea capitis - clinical and trichoscopic features. (A) Clinical photograph showing a patch of hair loss with overlying yellowish adherent scales and crusting. (B) Trichoscopy (polarised, 10×) of the same lesion demonstrating morse code hair (red arrow), a broken/dystrophic hair (blue arrow), a black dot (red circle), and perifollicular and interfollicular scaling (yellow arrow).

**Figure 2:**
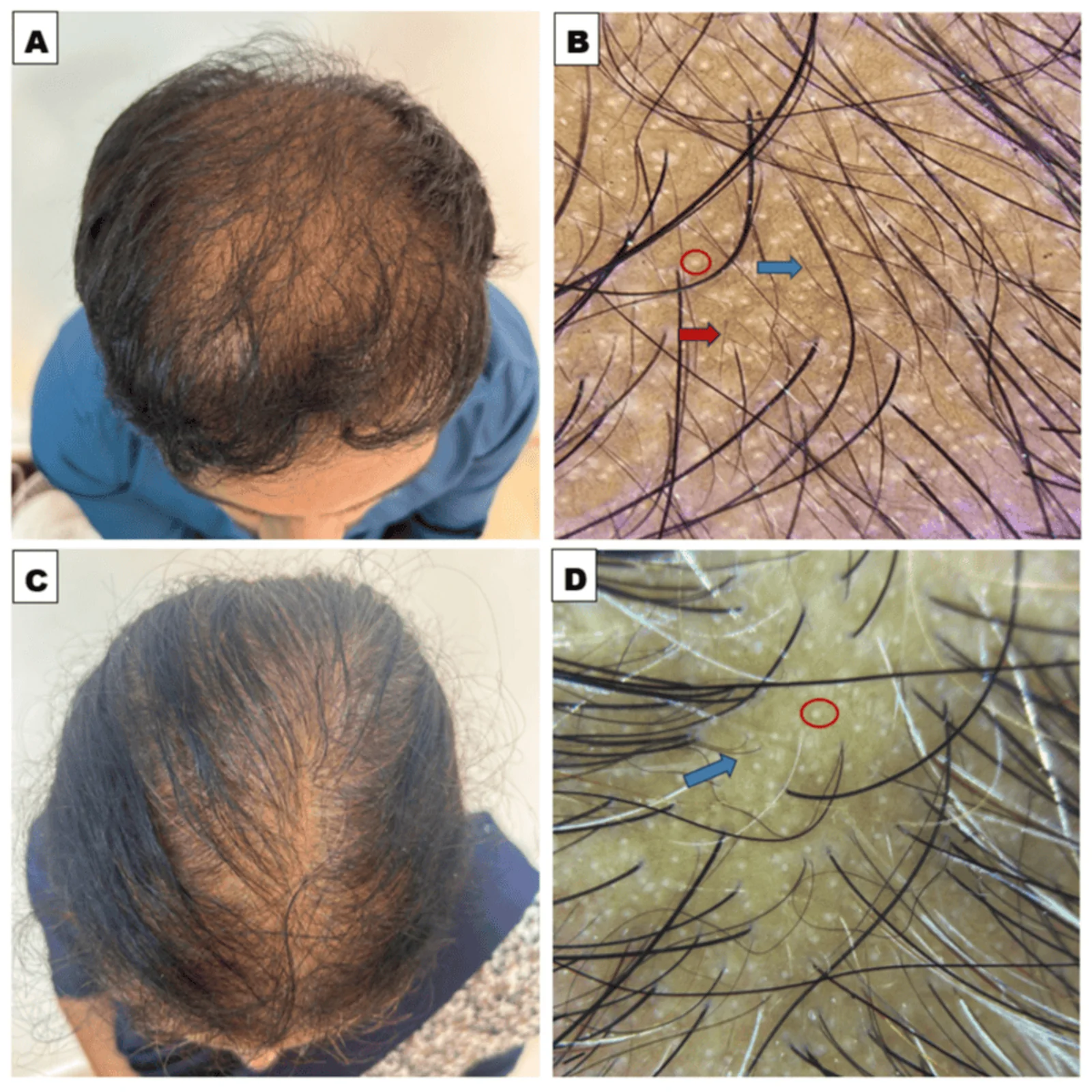
Patterned hair loss - male androgenetic alopecia (A, B) and female pattern hair loss (C, D). (A) Clinical photograph of male androgenetic alopecia showing frontovertical thinning. (B) Trichoscopy demonstrating miniaturised hair (blue arrow), empty follicles (red arrow) and short vellus hair (red circle). (C) Clinical photograph of female pattern hair loss showing diffuse central thinning with preservation of the frontal hairline. (D) Trichoscopy demonstrating empty follicles (red circle) and miniaturised hair (blue arrow).

**Figure 3:**
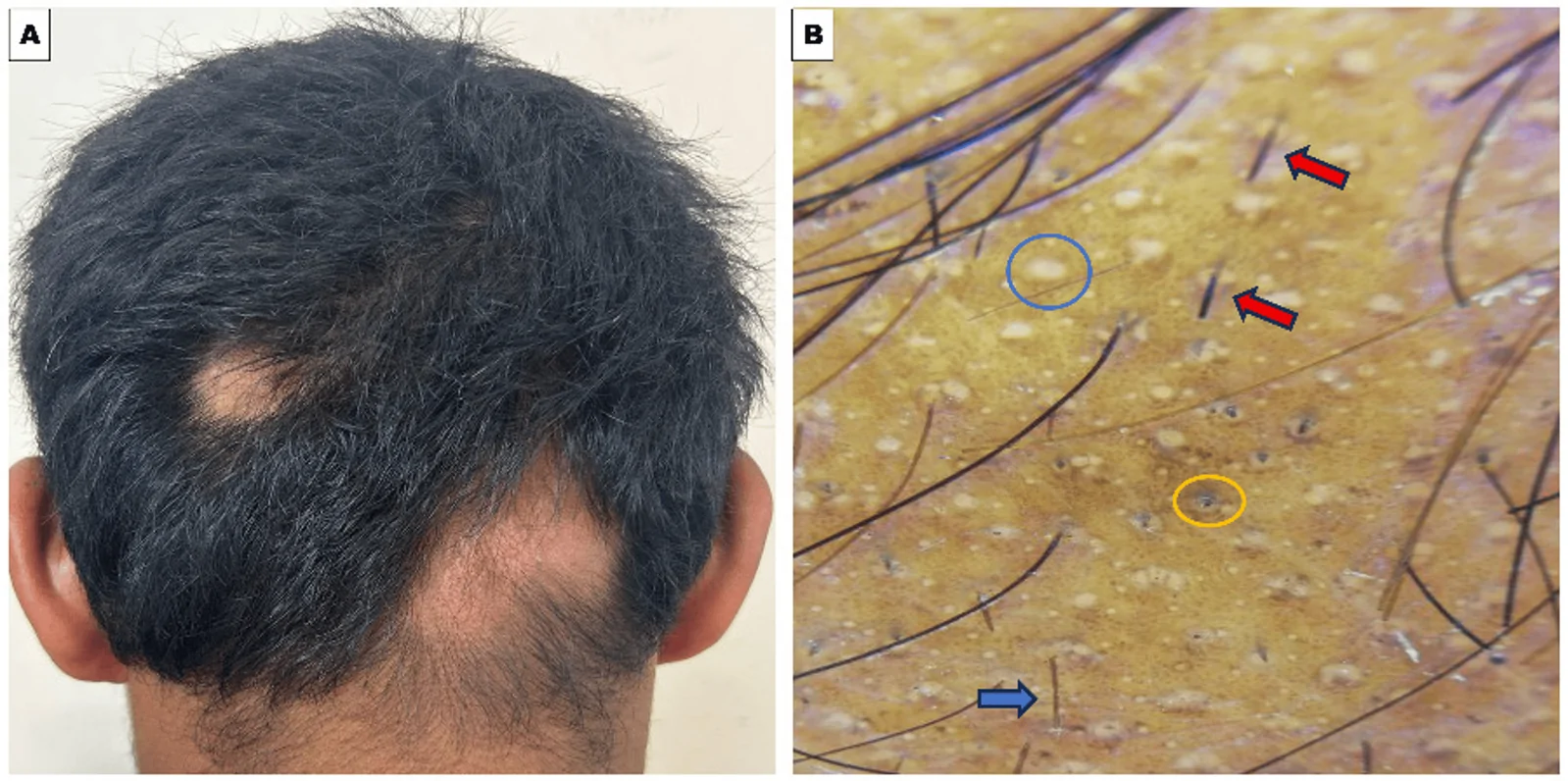
Alopecia areata - clinical and trichoscopic features. (A) Clinical photograph showing a well-circumscribed patch of non-scarring hair loss with a smooth scalp surface. (B) Trichoscopy (polarised, 10×) demonstrating exclamation mark hairs - tapering proximally (red arrows), black dots (yellow circle), a regularly distributed yellow dot (blue circle), and a broken hair (blue arrow). Exclamation mark hairs and black dots characterised alopecia areata in 33/40 (82.5%) and 34/40 (85.0%) of cases, respectively (p < 0.001), and distinguished it from its clinical look-alike, tinea capitis.

**Figure 4:**
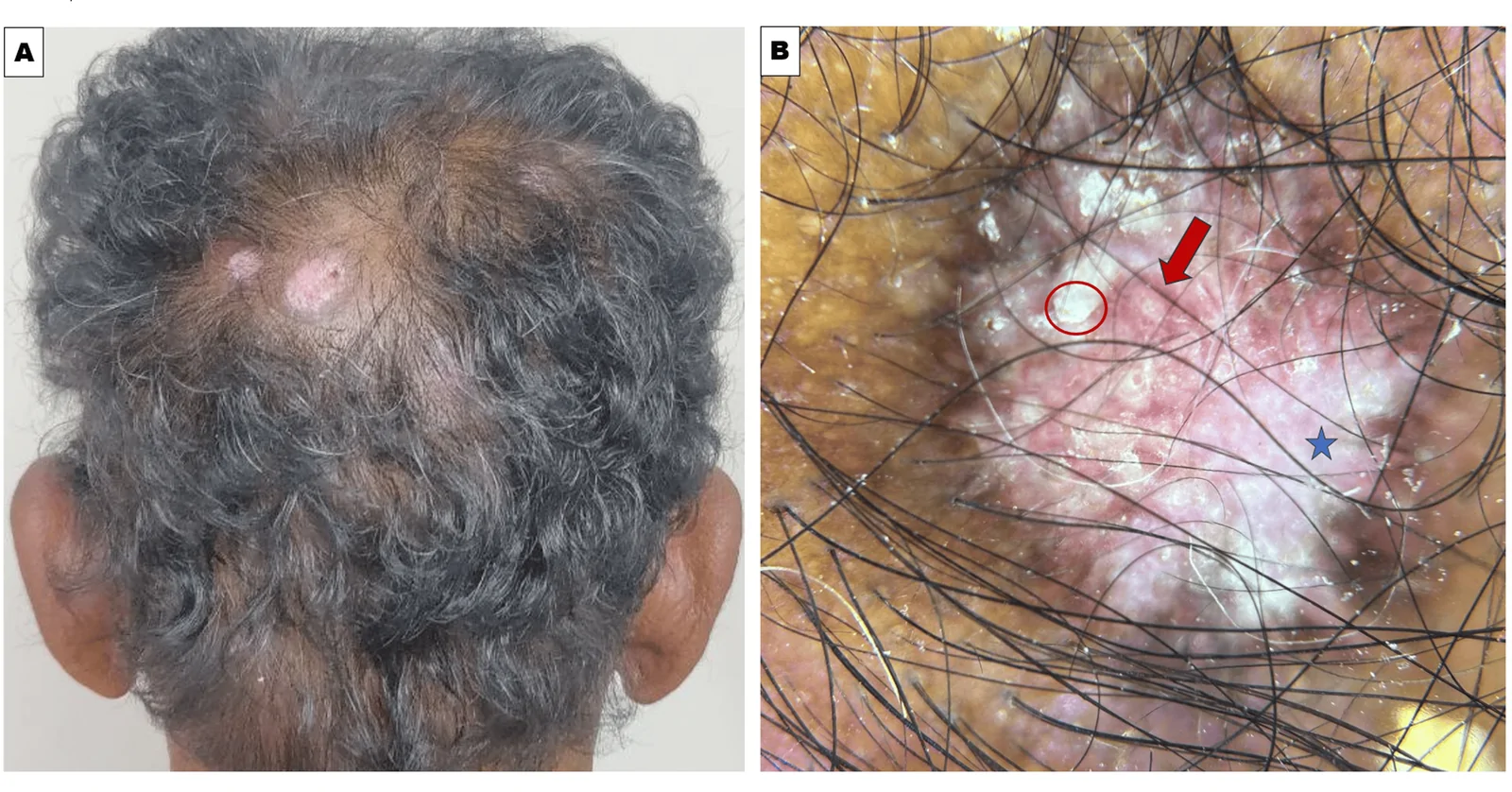
Cicatricial alopecia illustrated with discoid lupus erythematosus - clinical and trichoscopic features. (A) Clinical photograph showing patches of scarring hair loss with smooth, atrophic, ivory-white skin and loss of follicular markings. (B) Trichoscopy (polarised, 10×) demonstrating loss of follicular openings over a white structureless (fibrotic) background (blue star), keratotic follicular plugs (red circle) and perifollicular erythema/pigment disruption (red arrow). Loss of follicular openings was present in 7/7 (100%) of primary cicatricial alopecia cases and served as the unifying discriminator between scarring and non-scarring categories (p < 0.001); keratotic plugs and follicular red dots were the defining signature of discoid lupus erythematosus in this cohort.

Pairwise comparison across diagnostic categories confirmed the discriminatory value of several trichoscopic features (Table 3). Comma hairs were strongly associated with tinea capitis (11/14, 78.6%; p < 0.001; Figure 1). Hair diameter diversity >20% was the unifying feature of patterned hair loss, observed in 64/69 (92.8%) of male androgenetic alopecia and 30/34 (88.2%) of female pattern hair loss; yellow dots were present in 69/69 (100%) of male androgenetic alopecia versus 10/34 (29.4%) of female pattern hair loss (p < 0.001; Figure 2), reflecting prominent sebum- and keratin-filled dilated follicular infundibula overlying miniaturised or empty follicles, which are more conspicuous in male-pattern disease. Exclamation mark hairs and black dots characterised alopecia areata (33/40, 82.5%, and 34/40, 85.0%, respectively; p < 0.001; Figure 3), while loss of follicular openings was present in 7/7 (100%) of primary cicatricial alopecia (p < 0.001; Figure 4).

**Table 3:** Discriminatory trichoscopic features by diagnostic category. This table lists the hallmark trichoscopic feature for each diagnostic category, the number of patients showing that feature out of the category total (n/N), and the corresponding statistical significance. Comparisons were made across categories using the chi-square test, with a p-value < 0.05 considered significant; a dash indicates that significance testing was not applicable owing to small numbers. n: number of patients

Diagnostic category	Hallmark feature	n/N (%)	p-value
Male androgenetic alopecia (n = 69)	Yellow dots	69/69 (100.0)	< 0.001
Male androgenetic alopecia	Hair diameter diversity > 20%	64/69 (92.8)	< 0.001
Female pattern hair loss (n = 34)	Hair diameter diversity > 20%	30/34 (88.2)	< 0.001
Alopecia areata (n = 40)	Black dots	34/40 (85.0)	< 0.001
Alopecia areata	Exclamation mark hairs	33/40 (82.5)	< 0.001
Telogen effluvium (n = 30)	Short upright regrowing hairs	24/30 (80.0)	< 0.001
Tinea capitis (n = 14)	Comma hairs	11/14 (78.6)	< 0.001
Trichotillomania (n = 3)	Broken hairs of varied length	3/3 (100.0)	-
Cicatricial alopecia (n = 7)	Loss of follicular openings	7/7 (100.0)	< 0.001

Diagnostic accuracy of comma hairs for tinea capitis

Because comma hairs emerged as the trichoscopic feature with the largest diagnostic-pathway impact (responsible for both reclassified tinea capitis cases) and were not observed in any non-tinea diagnostic category in this cohort, a prespecified 2 × 2 analysis was performed against the final composite diagnosis of tinea capitis (Table 4). Of 14 patients with a final diagnosis of tinea capitis, 11 had comma hairs on trichoscopy (true positives), and three did not (false negatives). Of 186 patients without tinea capitis, none had comma hairs (no false positives), and all 186 were correctly classified (true negatives).

**Table 4:** 2 × 2 contingency table for comma hairs against the final diagnosis of tinea capitis (n = 200). This table cross-tabulates the presence or absence of comma hairs on trichoscopy against the final composite diagnosis of tinea capitis, providing the cell counts used to derive the test characteristics. TP: true positive; FP: false positive; FN: false negative; TN: true negative; n: number of patients

	Tinea capitis +	Tinea capitis -	Total
Comma hairs +	11 (TP)	0 (FP)	11
Comma hairs -	3 (FN)	186 (TN)	189
Total	14	186	200

Comma hairs demonstrated a sensitivity of 78.6% (11/14; 95% CI 52.4-92.4%) and a specificity of 100.0% (186/186; 95% CI 98.0-100.0%) for tinea capitis. Positive predictive value was 100.0% (11/11; 95% CI 74.1-100.0%), negative predictive value was 98.4% (186/189; 95% CI 95.4-99.5%), and overall diagnostic accuracy was 98.5% (197/200; 95% CI 95.7-99.5%). The negative likelihood ratio was 0.21, while the positive likelihood ratio was effectively infinite (no false positives observed); applying the rule-of-three to the upper bound of the false-positive proportion yielded a conservative lower bound for the positive likelihood ratio of 48.7. The association between comma hairs and tinea capitis was highly significant on Fisher's exact test (p < 0.001) (Table 5).

**Table 5:** Operating characteristics of comma hairs as a trichoscopic marker for tinea capitis. This table reports the diagnostic-test performance of comma hairs for tinea capitis, derived from the contingency data in Table 4. Confidence intervals for proportions were calculated using the Wilson score method, and the association was tested with Fisher's exact test. *No false-positive observations were recorded; the lower bound of the positive likelihood ratio was estimated by applying the rule-of-three to the upper 95% confidence bound of the false-positive proportion (3/186).

Diagnostic parameter	Estimate	95% CI
Sensitivity	78.6% (11/14)	52.4 - 92.4%
Specificity	100.0% (186/186)	98.0 - 100.0%
Positive predictive value	100.0% (11/11)	74.1 - 100.0%
Negative predictive value	98.4% (186/189)	95.4 - 99.5%
Diagnostic accuracy	98.5% (197/200)	95.7 - 99.5%
Positive likelihood ratio	∞ (lower bound 48.7)*	-
Negative likelihood ratio	0.21	-
Fisher’s exact p-value	< 0.001	-

Trichoscopy as a triage tool: impact on confirmatory investigations

Trichoscopy directed the use of confirmatory investigations rather than replacing them (Table 6). KOH examination was requested in 14 patients on the basis of comma or corkscrew hairs and returned positive in 12/14 (positive yield 85.7%); ANA was performed in two patients with trichoscopic features suggestive of discoid lupus erythematosus (1/2 positive); hormonal evaluation in 12 patients with female pattern hair loss and clinical hyperandrogenism clarified an overlap with chronic telogen effluvium in 1/12 cases. Scalp biopsy was performed in 8/200 patients (4.0%) but was the diagnosis-deciding investigation in only 2/200 (1.0%) - one lichen planopilaris case and one anagen effluvium case in which trichoscopy had been inconclusive; in the remaining six, biopsy confirmed an already trichoscopy-supported scarring diagnosis. This diagnosis-deciding rate is far below the biopsy rates traditionally cited for cicatricial and overlap alopecias evaluated without trichoscopy.

**Table 6:** Correlation of confirmatory investigations with the final diagnosis. This table shows how often each confirmatory investigation was performed, how often it returned an abnormal result, and whether it changed the final diagnosis, illustrating the role of trichoscopy in directing rather than replacing further testing. KOH: potassium hydroxide mount; ANA: antinuclear antibody; n: number of patients

Investigation	No. of patients tested (n)	Abnormal (n)	Diagnosis influenced?
Haemoglobin	18	9	No (supportive only)
Thyroid function tests	20	7	No (supportive only)
Hormonal evaluation	12	6	Yes - 1 overlap clarified
KOH examination	14	12	Yes - 2 cases reclassified
ANA	2	1	No (confirmatory)
Scalp biopsy	8	8	Diagnosis-deciding in 2 cases; confirmatory in 6

## Discussion

In this cross-sectional study of 200 patients evaluated in a rural tertiary care centre, trichoscopy contributed diagnostically in 195/200 (97.5%) of cases. This diagnostic yield is descriptively higher than the 90.5% conclusive yield reported by Chiramel et al. for difficult cases [11] and is broadly consistent with the utility described by Varma et al. [12]; however, given differences in patient selection, examiner training, and reference-standard methodology across these studies, this comparison is exploratory and hypothesis-generating rather than a validated head-to-head benchmark.

The single most clinically consequential finding of this study is the diagnostic-test performance of comma hairs for tinea capitis. The prespecified 2 × 2 analysis demonstrated a sensitivity of 78.6% (11/14) with a specificity and positive predictive value of 100% (186/186), and Fisher's exact p < 0.001 - figures that strongly support the bedside identification of comma hairs as a highly specific marker for tinea capitis, though these estimates must be interpreted with caution due to the study's structural limitations. The negative likelihood ratio of 0.21 indicates that the absence of comma hairs meaningfully reduces, though does not exclude, the probability of tinea capitis, consistent with the well-described variability of comma hairs across causative dermatophyte species. This high specificity is consistent with comma hairs being a well-established marker of tinea capitis, as confirmed in the systematic review by Waśkiel-Burnat et al. [13], and with the 85.7% prevalence reported by Chiramel et al. [11]. The finding operationally translates into the two reclassifications observed in our cohort: patients clinically diagnosed as alopecia areata were re-diagnosed as tinea capitis after comma hairs were identified, preventing "tinea incognito" through inadvertent corticosteroid therapy. Macroscopically, both conditions can present as patchy hair loss with black dots, and clinical examination alone is unreliable for distinguishing the two.

The remaining three reclassifications converted a diagnosis of telogen effluvium into female pattern hair loss based on hair diameter diversity >20% - a threshold that is virtually impossible to assess by naked-eye examination but is reliably visualised on trichoscopy. The redirection of management from reassurance to anti-androgen and minoxidil therapy in these patients illustrates how trichoscopy operationalises a treatment-relevant pathological criterion at the bedside. Although the 2.5% reclassification rate (5/200) is small in absolute terms, its clinical impact is disproportionately high.

Trichoscopy also operated as a triage tool that streamlined, rather than replaced, confirmatory investigation. KOH examination guided by trichoscopic clues yielded positive results in 12/14 (85.7%) of cases tested, and although scalp biopsy was performed in 8/200 (4.0%), it was the diagnosis-deciding investigation in only 2/200 (1.0%). This diagnosis-deciding rate is substantially lower than that traditionally reported in studies in which biopsy was the default investigation for all cicatricial and difficult non-cicatricial alopecias [11,14]. The reduction in invasive evaluation has implications for cost, patient acceptability, and access - particularly relevant in rural Indian populations where biopsy is often unavailable, declined for cultural reasons, or associated with logistical delays. However, trichoscopic interpretation is operator-dependent and requires appropriate training and experience, and the diagnostic performance observed in the present study reflects a single-centre setting with examiners familiar with the technique. Furthermore, the availability of even a basic handheld dermatoscope is a prerequisite for implementing this approach, and the costs associated with equipment acquisition were not evaluated in the present study. By converting microscopic markers into immediately interpretable visual data, trichoscopy compresses the diagnostic pathway and permits targeted therapy at the first visit. However, this advantage is inherently dependent on operator expertise, adequate training, and the availability of quality dermatoscopic equipment.

The 2.5% inconclusive rate (5/200) maps a clinically interpretable boundary for the technique. Three of the five inconclusive cases lay on the female pattern hair loss/chronic telogen effluvium spectrum, where early miniaturisation may not yet meet the 20% diameter-diversity threshold; one case of anagen effluvium overlapped with severe alopecia areata; and one cicatricial alopecia required histopathology to distinguish lichen planopilaris from another scarring process. These limits are consistent with the literature and reinforce that trichoscopy supplements but does not abolish the role of histopathology in advanced or overlapping cicatricial disease [9,10].

Beyond the headline yield, the discriminatory profile of individual signs aligned closely with established trichoscopic literature. Yellow dots were universal in male androgenetic alopecia (69/69, 100%) but markedly less common in female pattern hair loss (10/34, 29.4%; p < 0.001), supporting the interpretation that yellow dots in male androgenetic alopecia represent sebum-distended ostia of miniaturised follicles, whereas in female pattern hair loss, other markers - chiefly hair diameter diversity - drive the diagnosis [15]. Exclamation mark hairs in alopecia areata (33/40, 82.5%) were more frequent than the 18.6% reported by Govindarajulu et al. and the 45.9% reported by Varma et al. [12,16], a difference that probably reflects the predominance of active disease in our outpatient cohort and improved optical resolution of modern dermatoscopes. Universal loss of follicular openings (7/7, 100%) in cicatricial alopecia - irrespective of subtype - confirms its role as the unifying discriminator between scarring and non-scarring categories [17].

Several practical implications follow. In settings where trichoscopy is available, a structured trichoscopic protocol - searching first for loss of follicular openings (scarring vs non-scarring), then for hair diameter diversity (patterned vs non-patterned), then for pathognomonic shaft features (comma, exclamation mark, Pohl-Pinkus) - provides a high-yield decision tree at the bedside. In settings where trichoscopy is not yet available, our data argue for prioritising the acquisition of a handheld trichoscope as a high-impact, low-cost intervention.

This study has several limitations. It was a single-centre, hospital-based cross-sectional study; the population was therefore weighted towards patients with overt or advanced disease, and milder or transient alopecias may have been under-represented. The cicatricial alopecia subgroup was small (7/200, 3.5%), reflecting the true relative rarity of scarring alopecia in an unselected outpatient population. Consequently, the precision of subgroup estimates, including the apparent 100% sensitivity of loss of follicular openings, was limited, and these findings should be validated in larger, ideally multi-centre, cohorts of patients with cicatricial alopecia before being generalised. Trichoscopic interpretation was performed by clinicians at a single centre; inter-observer agreement was not formally assessed and warrants evaluation in future multi-centre studies. The cross-sectional design precludes longitudinal evaluation of trichoscopic features as biomarkers of treatment response. Trichoscopy was confined to scalp examination; non-scalp hair-bearing sites (eyebrows, beard, and body hair) were not systematically evaluated. Because trichoscopic findings were incorporated into the composite final diagnosis rather than being validated against a fully independent reference standard (e.g., histopathology in all cases), incorporation bias may have inflated the apparent sensitivity, specificity, and predictive values of individual trichoscopic signs, including comma hairs for tinea capitis. Future studies using an independent reference standard, interpreted blinded to trichoscopic findings, are required to provide unbiased estimates of these diagnostic performance characteristics.

## Conclusions

Trichoscopy proved to be a valuable, non-invasive first-line diagnostic tool in the evaluation of alopecia in a rural tertiary care setting. It enhanced diagnostic confidence, enabled clinically meaningful refinement of diagnoses in selected cases, facilitated targeted use of ancillary investigations, and reduced the need for invasive scalp biopsy. These findings support the integration of trichoscopy into routine dermatological practice, particularly in rural and resource-limited settings where rapid, accurate, and minimally invasive diagnosis is essential. Multi-centre studies incorporating formal assessment of inter-observer agreement and validation against an independent reference standard are needed to confirm these findings and to establish a standardised trichoscopic triage protocol for resource-limited settings.
